# Bioinformatic analyses of hydroxylated polybrominated diphenyl ethers toxicities on impairment of adrenocortical secretory function

**DOI:** 10.1265/ehpm.22-00023

**Published:** 2022-10-06

**Authors:** Zemin Cai, Wei Hu, Ruotong Wu, Shukai Zheng, Kusheng Wu

**Affiliations:** 1 Department of Preventive Medicine, Shantou University Medical College, Shantou 515041, Guangdong, China; 2 Chronic Disease Control Center of Shenzhen, Shenzhen 518020, Guangdong, China; 3 School of Life Science, Xiamen University, Xiamen 361102, Fujian, China

**Keywords:** Polybrominated diphenyl ethers, Bioaccumulation, Adrenocortical secretory function, Gene expression

## Abstract

**Background:**

Polybrominated diphenyl ethers (PBDEs) and their metabolites have severe impact on human health, but few studies focus on their nephrotoxicity. This study was conceived to explore hub genes that may be involved in two hydroxylated polybrominated diphenyl ethers toxicities on impairment of adrenocortical secretory function.

**Methods:**

Gene dataset was obtained from Gene Expression Omnibus (GEO). Principal component analysis and correlation analysis were used to classify the samples. Differentially expressed genes (DEGs) were screened using the limma package in RStudio (version 4.1.0). Gene Ontology (GO), Kyoto Encyclopedia of Genes and Genomes (KEGG) and Reactome enrichment analyses of DEGs were conducted. Protein-protein interaction (PPI) network was established using STRING network, and genes were filtered by Cytoscape (version 3.8.2). Finally, the hub genes were integrated by plug-in CytoHubba and RobustRankAggreg, and were preliminarily verified by the Comparative Toxicogenomics Database (CTD).

**Results:**

GSE8588 dataset was selected in this study. About 190 upregulated and 224 downregulated DEGs in 2-OH-BDE47 group, and 244 upregulated and 276 downregulated DEGs in 2-OH-BDE85 group. Functional enrichment analyses in the GO, KEGG and Reactome indicated the potential involvement of DEGs in endocrine metabolism, oxidative stress mechanisms, regulation of abnormal cell proliferation, apoptosis, DNA damage and repair. 2-OH-BDE85 is more cytotoxic in a dose-dependent manner than 2-OH-BDE47. A total of 98 hub genes were filtered, and 91 nodes and 359 edges composed the PPI network. Besides, 9 direct-acting genes were filtered for the intersection of hub genes by CTD.

**Conclusions:**

OH-PBDEs may induce H295R adrenocortical cancer cells in the disorder of endocrine metabolism, regulation of abnormal cell proliferation, DNA damage and repair. The screened hub genes may play an important role in this dysfunction.

**Supplementary information:**

The online version contains supplementary material available at https://doi.org/10.1265/ehpm.22-00023.

## 1. Background

Polybrominated diphenyl ethers (PBDEs), as brominated flame retardants, are widely used in various building materials, textiles and polymers for electronic equipment [1]. Lipophilic and persistent characteristics are the physicochemical properties of PBDEs, which make them bioaccumulated and amplified in the food chain and make severe threat to ecosystems and human health [2–4]. PBDEs have been detected in blood, adipose, hair, placenta, breast milk, kidney, liver, lung and semen and other tissues in humans [5–7]. Besides, their toxicities are connected to endocrine disruption, reproductive toxicity, developmental neurotoxicity and immunotoxicity [8, 9].

PBDEs and their metabolites are proved to have toxicity to humans, but the mechanism is still unclear. On account of the intricate mechanism of PBDEs toxicity, behavioral, transcriptomic, metabolomic, and other approaches are often combined to explore the mechanisms. For example, the zebrafish model was used to explore the neurodevelopmental toxicity of PBDEs via a combination of behavioral transformation evaluation and transcriptomics [10]. Previous studies [11, 12] have showed that PBDE concentrations and altered hormone milieu have raised the risk of female reproductive outcomes and fetal growth.

The study [13] showed that PBDEs, known as one of endocrine disruptors and neurotoxins, might affect the steroid enzymes responsible for various steroid hormone biosynthesis and ultimately lead to reproductive conditions, cancer and other pathological problems associated with growth and development. Since PBDEs are structurally similar to endogenous thyroid hormone, they primarily target the thyroid system [14]. Thyroid hormones gradually fade away for high doses of PBDEs, and bind to thyroid hormone receptors and transporter proteins [15–17]. Furthermore, exposure to endocrine disruptors is associated with cancer risk in various tissues, and the activation of environmental estrogen fast signals can result in epigenetic reprogramming and develop to breast carcinoma [18].

The study [19] found that oxidative damage induced by PBDEs has been found to be a potential mechanism of nephrotoxicity and hepatotoxicity. Previous work [13] has shown that PBDEs and their metabolites have influence on adrenosteroid production in H295R adrenocortical cancer cells, but the study on their toxicities and thermal decomposition is restricted. BDE-47 and BDE-85 are the two congeners of PBDEs [20]. BDE-47 is one of the most common congeners, but has not been examined for carcinogenicity in model systems [21]. Another congener BDE85 has a similar structure to 2,3,7,8-tetrachlorodibenzo-p-dioxin (TCDD) and affinity for the aryl hydrocarbon receptors (AHR) [22]. After PBDEs undergoing phase I metabolism, hydroxylated polybrominated diphenyl ethers (OH-PBDEs) metabolites formed in animals, which may cause more severe biological effects [23]. OH-PBDEs are new type of organic pollutant, and have gradually become a new hotspot for environmental research field [24, 25]. Besides, the study indicated that 2-OH-BDE85 has stronger dose-dependent toxicity than 2-OH-BDE47 [26].

In recent years, with the development of various high throughput technologies, high throughput screening for toxicological relationships has been possible through genome analysis of the bioinformatics, rapid mapping of biological pathways and genes involved in toxicological interference, and effective analysis of mechanisms and pathways [27–31]. Global transcriptome sequencing analysis (RNA-seq) can effectively discern potential biomarkers, filter related biopathways, and help us probe the potential toxic mechanism [32].

Based on the transcriptome data, a bioinformatics method was used in this study to explore hub genes for two hydroxylated polybrominated diphenyl ethers (2-OH-BDE47 and 2-OH-BDE85) disrupting effects on the secretion function of adrenal cortex. This study provides a scientific basis for further study of the toxicological mechanism of endocrine and metabolic disorders of PBDEs.

## 2. Methods

### 2.1 Collection of Microarray datasets

The Gene Expression Omnibus (GEO, https://www.ncbi.nlm.nih.gov/geo) database was selected to explore all datasets that have assessed the microarray data in PBDEs and adrenocortical carcinoma. The search details (“polybrominated diphenyl ethers” OR “PBDEs”) AND (“adrenocortical carcinoma” OR “adrenocortical cancer”) were searched in the medical subject headings (MeSH).

The Microarray dataset GSE8588 meets the retrieval requirements. Affymetrix Human Genome U133 Plus 2.0 Array (base on Affymetrix GPL570 platform) was used to detect gene expression. Microarray technology was used to analyze the gene expression in H295R adrenocortical cancer cells which was exposed to OH-PBDEs. After 24 hours respectively induced by 10 µM of 2-OH-BDE47 or 2-OH-BDE85, the cells were observed for the gene expression changes of OH-PBDE-induction. Finally, the experiments subdivide to control, 2-OH-BDE47 and 2-OH-BDE85 samples. The entry type of the dataset GSE8588 is Samples, corresponding 9 GEO Sample (GSM) ID. Each GSM accession correspond 54, 675 genes. The biomaterial provider is American Type Culture Collection, and the organism of the dataset is homo sapiens.

### 2.2 Identification of differentially expressed genes

RStudio (version 4.1.0) was used to disposal and standardize the data. After downloading the matrix data, the quantile standardization method was used to standardize the gene expression matrix among groups (Supplementary Figure 1S). According to the similarity of gene expression models in diverse samples, principal component analysis (PCA) and correlation analysis were used to classify nine samples into three groups, including control group, 2-OH-BDE47 group and 2-OH-BDE85 group, then PCA plot and correlation heatmap were visualized. The limma [33] was used to explore the significant differentially expressed RNAs in samples from cells with 2-OH-BDE47 or 2-OH-BDE85 and normal samples. The false discovery rate (FDR) was used to calculate the statistical significance of multiple inspection. Genes with FDR <0.05 and Log_2_[Fold Change] (Log_2_[FC]) > 1 were considered as DEGs and were visualized through volcano plot.

### 2.3 Signaling pathway enrichment analysis

Gene Ontology (GO) and Kyoto Encyclopedia of Genes and Genomes (KEGG) analysis of the significant DEGs were analyzed by the clusterProfiler [34] package, which used to compare biological subject between gene clusters. GO is the main bioinformatics method for annotating genes and analyzing the biological process [35], and is used to describe three types of gene functions: biological process (BP), cellular component (CC), and molecular function (MF). KEGG database is a comprehensive database for the systematic analysis of the metabolic pathways of gene products and compounds in cells and the function of these gene products for functional annotation of the genome or transcriptome of species [36]. The GO and KEGG analyses associated with adjusted *P*-value < 0.05 were considered to be statistically significant (Supplementary Table 1S and 2S).

The ReactomePA [37] is an R package for reactome pathway and visualization of the significant DEGs. Reactome [38] is an opensource relational database of signals and metabolic molecules that contains information about signal transduction, transport, DNA replication, metabolism, and other transport related molecular networks. The Reactome enrichment analysis associated with adjusted *P*-value < 0.05 was considered to be significant.

### 2.4 Identification of hub genes in regulation network

STRING (https://string-db.org/) is a biological network database of protein interactions [39]. The protein-protein interaction (PPI) of DEGs-encoded proteins was demonstrated by STRING (version 11.0), by searching limitation of “Homo sapiens” and a score >0.900 in accord with high confidence interaction as significant value, and the unconnected nodes in the network were hidden. PPI network construction was conducted by Cytoscape (version 3.8.2). Plug-in CytoHubba [40] was used to identify hub genes and sub-networks from complex interaction group. CytoHubba algorithms include Betweenness, BottleNeck, Closeness, Clustering Coefficient, Degree, DMNC, EcCentricity, EPC, MCC, MNC, Radiality and Stress algorithms. The RobustRankAggreg, an R package for robust rank aggregation with the criteria of adjusting *P* value < 0.05, was used to integrate and rank the algorithms. The hub genes were then filtered and their interactions were integrated by Cytoscape (version 3.8.2). The Comparative Toxicogenomics Database (CTD, http://ctdbase.org/) [41] was used to identify co-interacting genes of two hydroxylated polybrominated diphenyl ethers toxicities, to preliminarily verify the hub genes.

## 3. Results

### 3.1 Identification of differentially expressed genes

**Fig. 1:**
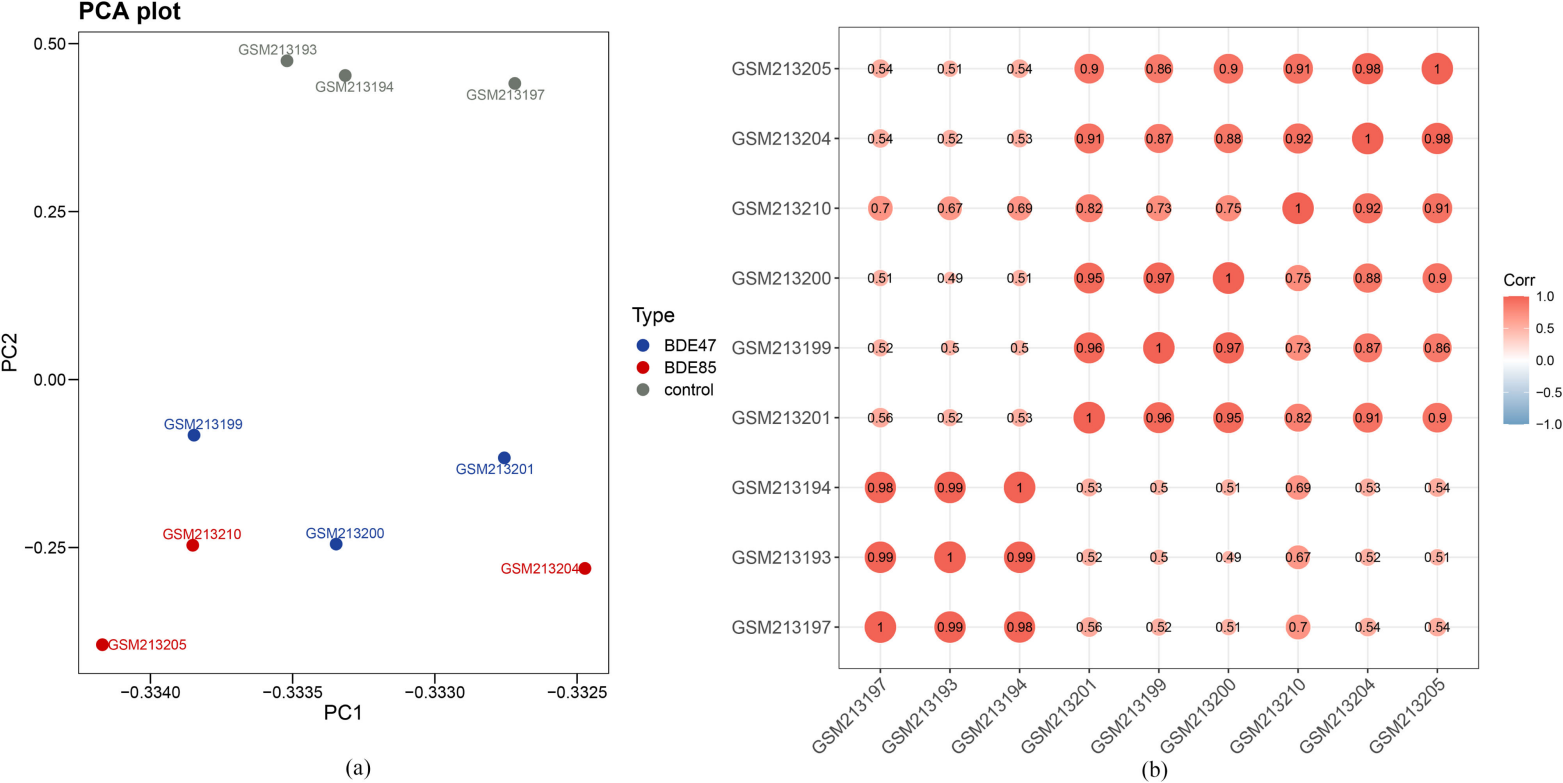
The PCA plot and correlation heatmap. (a) Principal component analysis of GSE8588. Gray dots represent control group, blue dots represent 2-OH-BDE47 group, and red dots represent 2-OH-BDE85 group. (b) The correlation analysis of GSE8588. Red spots represent positive correlation, and blue spots represent negative correlation. The numbers in each bar represent the correlation coefficient.

**Fig. 2:**
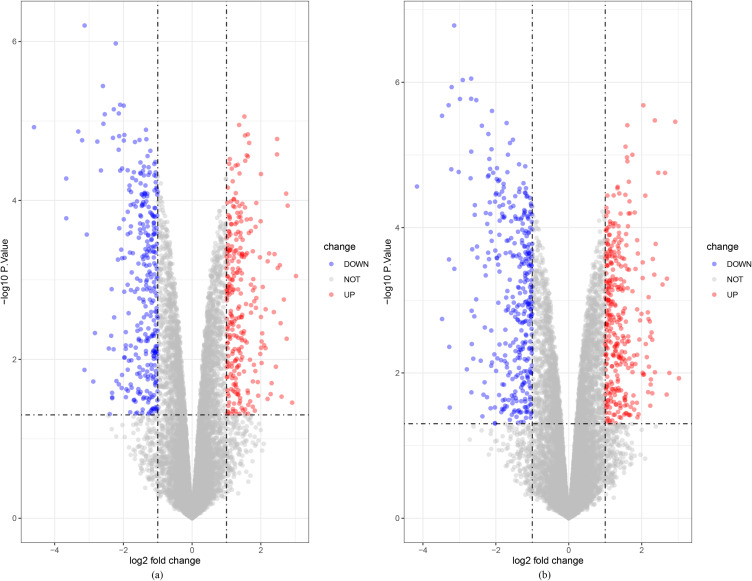
The volcano plot of significant DEGs. (a) Significant DEGs of 2-OH-BDE47 group. (b) Significant DEGs of 2-OH-BDE85 group. Red dots represent upregulated DEGs, and blue dots represent downregulated DEGs.

The Microarray datasets (GSE8588) was included in the study. PCA and correlation analysis showed that the gene expression patterns of the 2-OH-BDE47 group and 2-OH-BDE85 group had significant correlation (r range from 0.73 to 0.98, *P* < 0.01). The two groups were weakly correlated with control group (Fig. 1a, Fig. 1b). About 603 significant DEGs (278 upregulated and 325 downregulated DEGs) were found in 2-OH-BDE47 group, and 705 significant DEGs (339 upregulated and 366 downregulated DEGs) were found in 2-OH-BDE85 group (Fig. 2).

### 3.2 Signaling pathway enrichment in DEGs

**Fig. 3:**
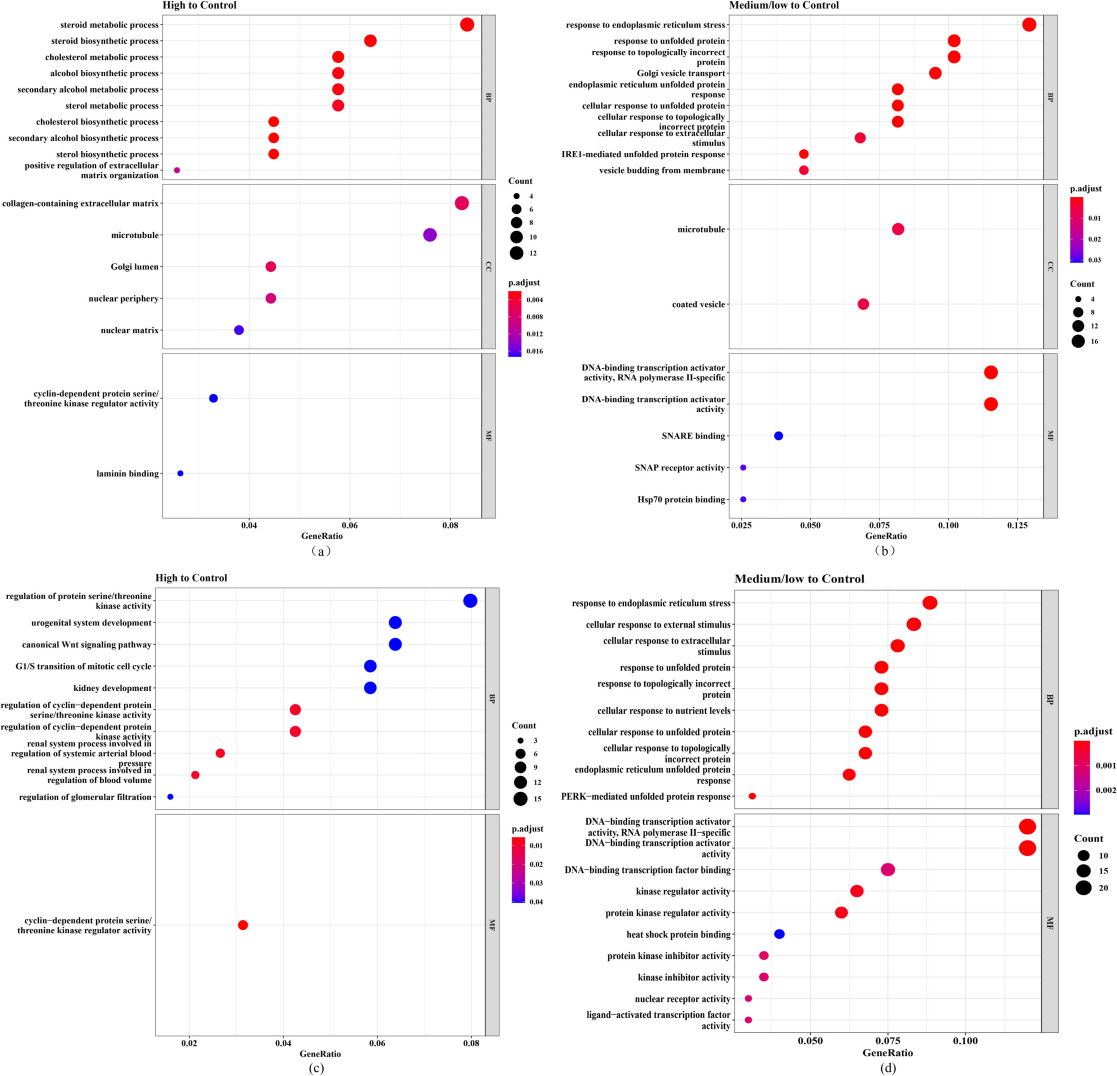
The GO analyses for significant DEGs. The bubble charts present GO analysis results of (a) upregulated DEGs and (b) downregulated DEGs in 2-OH-BDE47 group, (c) upregulated DEGs and (d) downregulated DEGs in 2-OH-BDE85 group.

The significant upregulated and downregulated DEGs were utilized for GO and KEGG analyses. A total of 190 upregulated and 224 downregulated DEGs in 2-OH-BDE47 group, and 244 upregulated and 276 downregulated DEGs in 2-OH-BDE85 group. For GO analysis of 2-OH-BDE47 in the GSE8588 dataset, the biological processes of the upregulated DEGs were mainly involved in steroid metabolic process, steroid biosynthetic process and cholesterol metabolic process (Fig. 3a); the downregulated DEGs were mainly involved in response to endoplasmic reticulum stress, response to unfolded protein and response to topologically incorrect protein (Fig. 3b). The cellular components of the upregulated DEGs were involved in collagen-containing extracellular matrix, microtubule and Golgi lumen (Fig. 3a); the downregulated DEGs were involved in microtubule and coated vesicle (Fig. 3b). The molecular functions of the upregulated DEGs were involved in cyclin-dependent protein serine/threonine kinase regulator activity and laminin binding (Fig. 3a); the downregulated DEGs were involved in DNA-binding transcription activator activity, RNA polymerase II-specific and DNA-binding transcription activator activity (Fig. 3b).

In the GO analysis of 2-OH-BDE85 group in the GSE8588 dataset, the biological processes of the upregulated DEGs were mainly involved in regulation of cyclin–dependent protein serine/threonine kinase activity, regulation of cyclin–dependent protein kinase activity and renal system process involved in regulation of systemic arterial blood pressure (Fig. 3c); the downregulated DEGs were mainly involved in response to endoplasmic reticulum stress, cellular response to external stimulus and cellular response to extracellular stimulus (Fig. 3d). The molecular functions of the upregulated DEGs were involved in cyclin-dependent protein serine/threonine kinase regulator activity (Fig. 3c); the downregulated DEGs were involved in DNA-binding transcription activator activity, RNA polymerase II-specific, DNA-binding transcription activator activity and DNA-binding transcription factor binding (Fig. 3d).

**Fig. 4:**
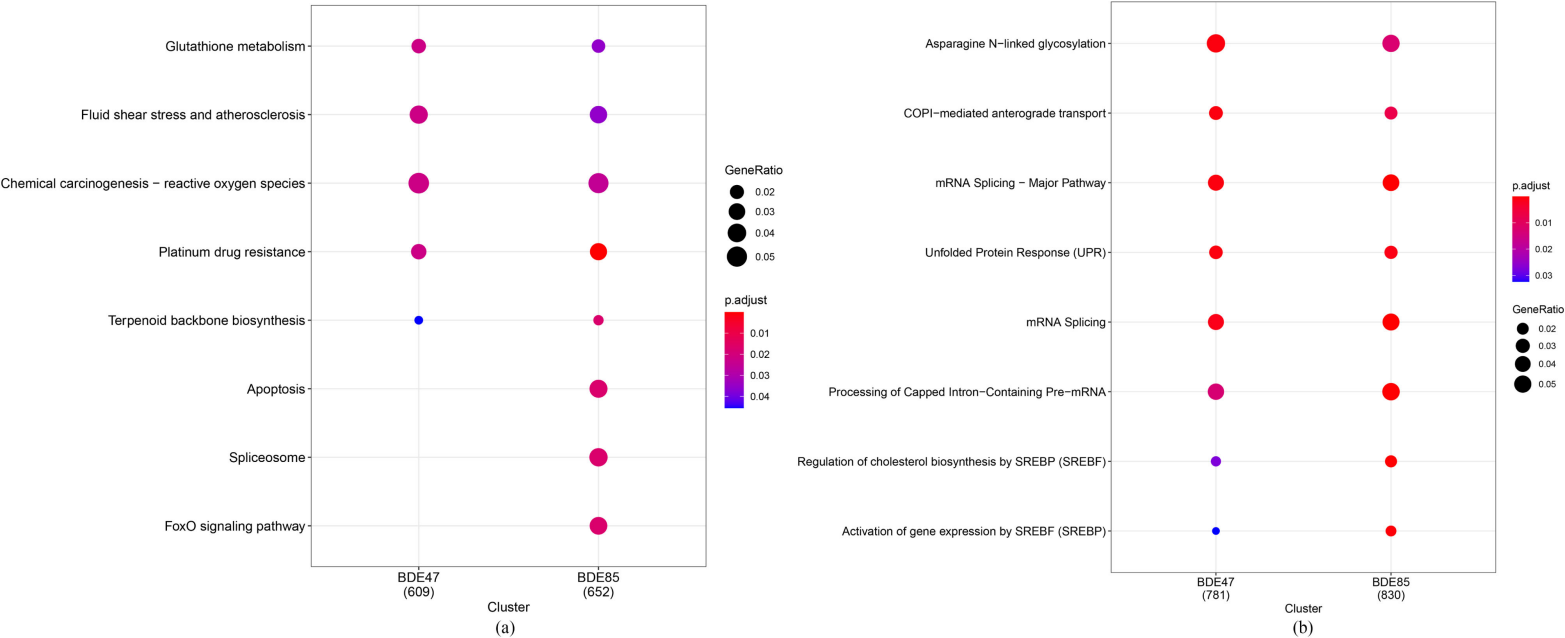
The KEGG and Reactome enrichment analyses for significant DEGs.

The KEGG pathway enrichment analysis showed that DEGs in 2-OH-BDE47 group and control group were enriched in 5 pathways, including chemical carcinogenesis-reactive oxygen species, fluid shear stress and atherosclerosis and glutathione metabolism (Fig. 4a); DEGs in 2-OH-BDE85 group and control group were enriched in 8 pathways, mainly including chemical carcinogenesis-reactive oxygen species, apoptosis and spliceosome (Fig. 4a).

The Reactome pathway enrichment analysis showed that DEGs in 2-OH-BDE47 group and 2-OH-BDE85 group were enriched in 8 pathways, mainly including asparagine N–linked glycosylation, Unfolded Protein Response (UPR) and mRNA Splicing (Fig. 4b).

### 3.3 Identification of hubgenes in regulation network

**Fig. 5:**
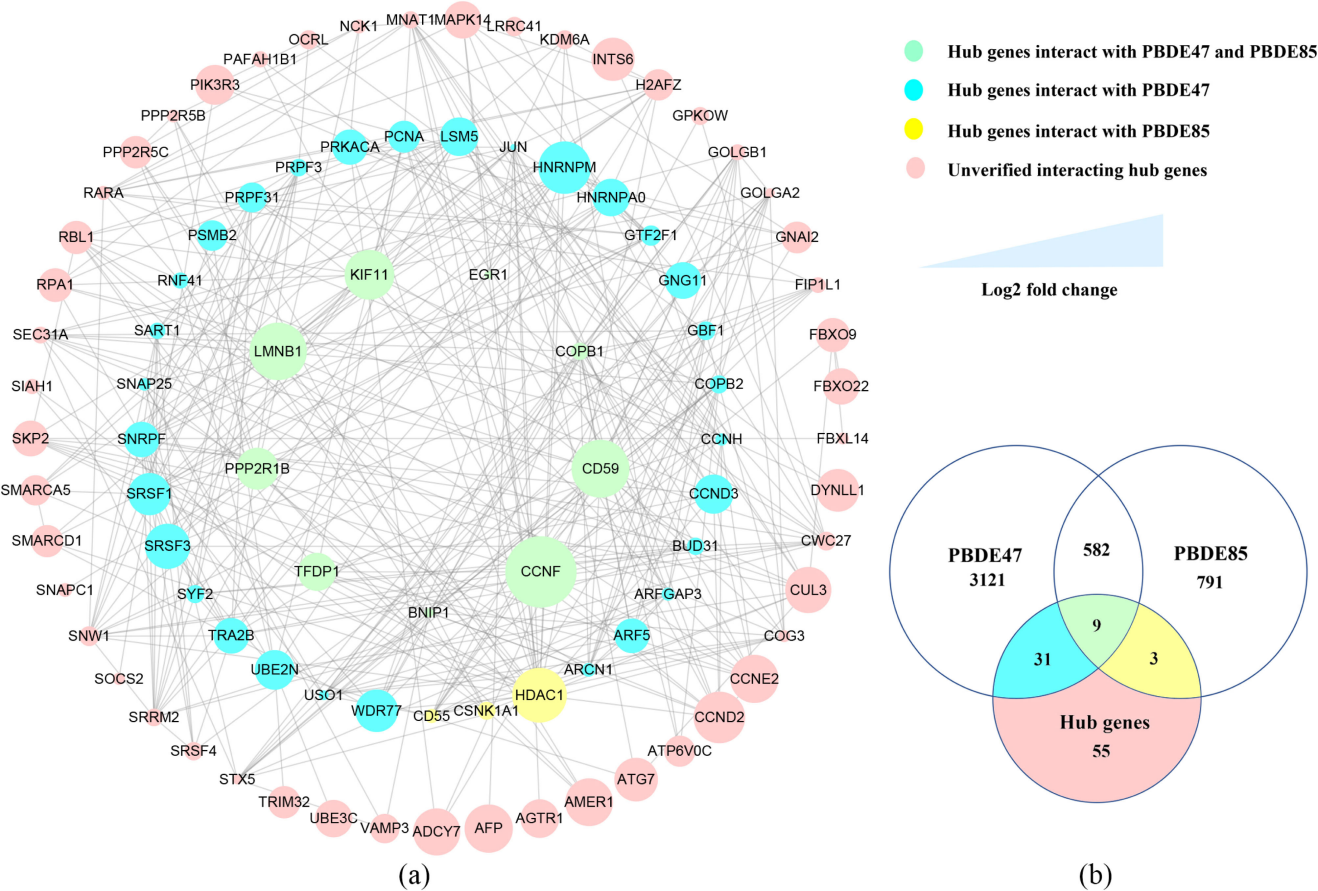
The PPI network and venn plot of hub genes (a) The PPI network of hub genes consists of 91 nodes and 359 edges. The nodes’ sizes represent the log2FC of hub genes. (b) Venn plot of hub genes. The nodes’ colors represent different hub genes: green nodes represent hub genes interact with PBDE47 and PBDE85 based on CTD, blue nodes and yellow nodes respectively represent hub genes interacting with PBDE47 and PBDE85, pink nodes represent unverified interacting hub genes.

In the 2-OH-BDE47 group, 1289 DEGs were uploaded to STRING, among which 706 DEGs were successfully mapped. The PPI network of 2-OH-BDE47 group was composed of 706 nodes and 3075 edges by Cytoscape. In the 2-OH-BDE85 group, 1457 DEGs were uploaded to STRING, among which 1201 DEGs were successfully mapped. The PPI network of 2-OH-BDE85 group was consist of 1201 nodes and 8970 edges (Supplementary Figure 2S). MCC, MNC, Degree, EPC and EcCentricity of CytoHubba algorithms were used to integrate and rank the hub genes. A total of 98 hub genes were filtered after taking the intersection of 2-OH-BDE47 group and 2-OH-BDE85 group by using a composite score of <0.05 as a criterion, then they were uploaded to STRING, among which 91 were successfully mapped by STRING. The PPI network was composed of 91 nodes and 359 edges through Cytoscape (Fig. 5a). Besides, the co-interacting genes of two hydroxylated polybrominated diphenyl ethers toxicities were identified by 591 genes after searching CTD. About 9 direct-acting genes (COPB1, CD59, PPP2R1B, KIF11, CCNF, BNIP1, TFDP1, EGR1 and LMNB1) were filtered for the intersection of hub genes and CTD result. 31 hub genes are only associated with PBDE47 and 3 hub genes (HDAC1, CD55, CSNK1A1) with PBDE85 (Fig. 5b), and their interactions were showed in Fig. 5a.

## 4. Discussion

PBDEs and adrenocortical carcinoma microarray data were retrieved in the GEO database. The Microarray dataset GSE8588 was meet the transcriptome expression profile. The dataset was provided by University of Leicester, to explore whether the accumulation of OH-PBDE will lead to the impairment of adrenal cortex secretion function [13]. In this study, DEGs of human adrenocortical carcinoma were screened by bioinformatics, and the biological effects of DEGs were explored by GO, KEGG and Reactome enrichment analyses. Then the PPI network screening genes were constructed based on DEGs to find the hub genes of OH-PBDE on renal metabolic toxicity.

The gene expression transformation induced by 2-OH-PBDE47 could be sorted as several significantly enriched GO biological processes: cholesterol metabolic and biosynthetic process, response to endoplasmic reticulum stress and response to unfolded protein. Besides, the gene expression transformation induced by 2-OH-PBDE85 could be sorted as several significantly enriched GO biological processes: apoptosis, cell cycle, transcription, organic acid metabolism and urogenital system development. Previous studies [42, 43] conducted that OH-PBDEs were found to have significant effects on steroid enzymes such as CYP17 and CYP19 in human adrenocortical carcinoma (H295R) cells line. As endocrine disruptors, OH-PBDEs have the potential to interfere with steroid production and derivatives [44], which is consistent with our study. In this study, the cytotoxicity of H295R cells mediated by OH-PBDEs is associated with the induction of genes involved in the endoplasmic reticulum stress response, such as mediate correct protein folding, cell cycle arrest and cell death/apoptosis. Overaccumulation of unfolded proteins in the endoplasmic reticulum during the biosynthesis of secretory proteins expressed by adrenal cortex cells results in endoplasmic reticulum stress. In order to alleviate endoplasmic reticulum stress, mammalian cells activate unfolded protein response, inhibit translation to prevent further accumulation of unfolded proteins, and induce transcription of endoplasmic reticulum chaperone and endoplasmic reticulum related degradation component genes. To some degree, apoptotic cells may be induced to die in order to safely dispose of damaged cells [45]. Moreover, OH-PBDEs significantly aggrandized the expression of stress response, pro-apoptotic and cell cycle arrest genes, which may account for more severe toxicity of 2-OH-BDE85 [13].

Furthermore, KEGG enriched analysis showed that both 2-OH-BDE47 and 2-OH-BDE85 were mainly enriched in endocrine regulation, fluid shear stress and atherosclerosis and chemical carcinogenesis-reactive oxygen species, and 2-OH-PBDE85 enrich in more pathways, including apoptosis, spliceosome and FoxO signaling pathway. The biological pathways of KEGG mainly include endocrine metabolism, genetic information processing, environmental information processing, cell processes and biological systems. 2-OH-BDE85 increases apoptosis and express higher cytotoxicity, which is consistent with the GO enriched analysis. Similar results have been found in the transcriptomics and metabolomics of human bronchial epithelial cells [46] and human neuroblastoma cells [47]. PBDEs not only interfere with oxidative stress mechanisms, but also affect the regulation of abnormal cell proliferation, apoptosis, DNA damage and repair. The Reactome pathway enrichment analysis showed that asparagine N–linked glycosylation, Unfolded Protein Response (UPR) and mRNA Splicing were the main pathways of two hydroxylated polybrominated diphenyl ethers toxicities. The biological pathways in Reactome include intermediate metabolism, signal transduction, transcriptional regulation and apoptosis. The central field of the Reactome data model is the reaction, which form a network of biological interactions and are classified into pathways [48].

In order to find out the degree of interaction between genes, two PPI networks were constructed by Cytoscape to furtherly confirm the hub genes. And hub genes were explored by CytoHubba algorithms [40] and RobustRankAggreg package in R. A total of 98 were filtered and uploaded to STRING and finally 91 hub genes were mapped. 43 direct-acting genes and 55 indirect-acting genes were intersected based on CTD. OH-PBDEs are structurally similar to thyroxine and triiodo-thyronine. They exhibit similar characteristics to thyroid hormones (THs), disrupt endocrine homeostasis, and compete with THs in binding to thyroid hormone transport proteins, transthyretin as well as thyroxine-binding globulin [15]. OH-PBDEs induce endoplasmic reticulum (ER) stress by key regulatory factors such as ER chaperones and ER transmembrane proteins, which can perceive the accumulation of unfolded proteins [49]. The genes are also induced and express by ER stress, such as stress-responsive, pro-apoptotic and cell cycle arrest genes [50]. Moreover, the expression of apoptosis/cell death induced by 2-OH-BDE85 is more significant, which may be the reason of the high cytotoxicity of 2-OH-BDE85 [13].

### Limitation and strength

The study has some limitations. Firstly, the study selected one dataset to explore the PBDEs on impairment of adrenocortical secretory function for the reason that only one dataset meets the retrieval requirements. Secondly, there is no experimental evidence for the interactions between hub genes network, which should be verified experimentally to improve reliability in our future research. Despite these limitations, high throughput screening for toxicological relationships has become a new tendency through genome analysis of the bioinformatics, rapid mapping of biological pathways and genes involved in toxicological interference, and effective analysis of mechanisms and pathways. Thus, the results provide evidence that hub genes network can affect the occurrence and development of impairment of adrenocortical secretory function by regulating the activity and the expression of hub genes.

## 5. Conclusions

The study was designed to explore hub genes that may be involved in two hydroxylated polybrominated diphenyl ethers toxicities on impairment of adrenocortical secretory function. A total of 1289 DEGs in 2-OH-BDE47, 1457 DEGs in 2-OH-BDE85 were identified and finally 98 hub genes were filtered. When exposed to specific doses of OH-PBDEs, DEGs may be involved in adrenal cortex endocrine dysfunction, abnormal regulation of cell proliferation, apoptosis, DNA damage and repair, in which the hub gene plays an important role.

## Abbreviations

PBDEs: Polybrominated diphenyl ethers
GEO: Gene Expression Omnibus
DEGs: Differentially expressed genes
GO: Gene Ontology
KEGG: Kyoto Encyclopedia of Genes and Genomes
PPI: Protein-protein interaction
CTD: Comparative Toxicogenomics Database
TCDD: 2,3,7,8-tetrachlorodibenzo-p-dioxin
AHR: Aryl hydrocarbon receptors
OH-PBDEs: Hydroxylated polybrominated diphenyl ethers
MeSH: Medical subject headings
PCA: Principal component analysis
FDR: False discovery rate
Log2[FC]: Log2[Fold Change]
UPR: Unfolded Protein Response
THs: Thyroid hormones
ER: endoplasmic reticulum

## Supplementary information

**Additional file 1:** **Figure 1S** Standardizing comparison of significant DEGs. (a) Box plot before standardizing the DEGs. (b) Box plot after standardizing the DEGs. **Figure 2S** PPI networks of DEGs. (a) 706 DEGs of 2-OH-BDE47 group were mapped, including 706 nodes and 3075 edges. (b) 1457 DEGs of 2-OH-BDE85 group were mapped, including 1201 nodes and 8970 edges. **Table 1S** Basic information of 2-OH-BDE47 DEGs in KEGG pathway. **Table 2S** Basic information of 2-OH-BDE85 DEGs in KEGG pathway.

## Declarations

### Availability of data and material

The datasets of PBDEs and their derivatives affect adrenal steroidogenesis in H295R adrenocortical cancer cells were acquired from GEO database, please visit: https://www.ncbi.nlm.nih.gov/geo.
