# Supplementary material for: Bioinformatic analyses of hydroxylated polybrominated diphenyl ethers toxicities on impairment of adrenocortical secretory function
Source: Environ Health Prev Med. 2022 Oct 6;27:38. doi: 10.1265/ehpm.22-00023 (PMC9556975; doi:10.1265/ehpm.22-00023)
Supplement: Supplementary file 1 — Additional file 1: Figure 1S Standardizing comparison of significant DEGs. (a) Box plot before standardizing the DEGs. (b) Box plot after standardizing the DEGs. Figure 2S PPI networks of DEGs. (a) 706 DEGs of 2-OH-BDE47 group were mapped, including 706 nodes and 3075 edges. (b) 1457 DEGs of 2-OH-BDE85 group were mapped, including 1201 nodes and 8970 edges. Table 1S Basic information of 2-OH-BDE47 DEGs in KEGG pathway. Table 2S Basic information of 2-OH-BDE85 DEGs in KEGG pathway. [file ehpm-27-038-s001.pdf]

Supplementary materials

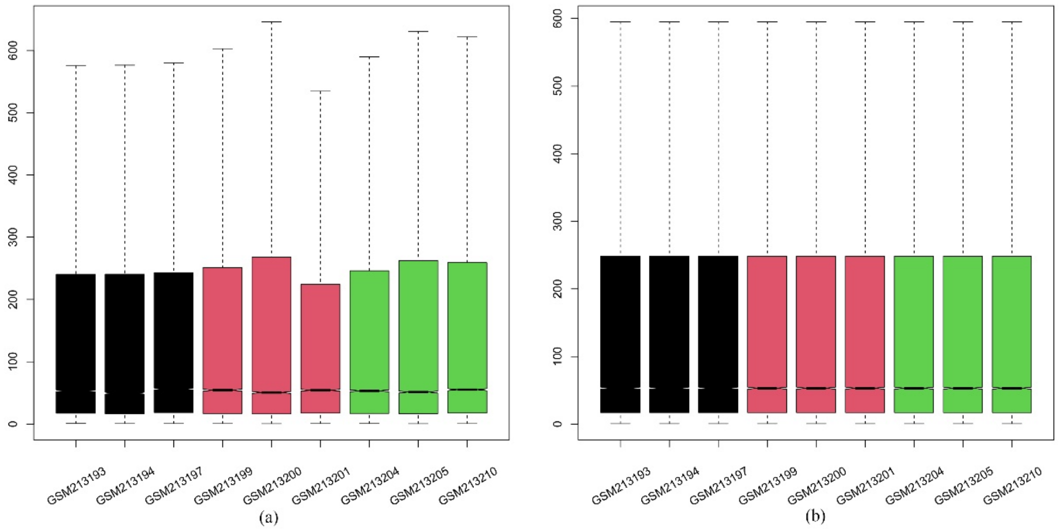

Figure 1S

Table 1S

ID	Pathways	Genes
hsa05418	Fluid shear stress and atherosclerosis	GPC1/SDC1/MAP2K6/BCL2/MGST2/PIK3R3/GSTT2/MAPK14/GSTM4/GSTA1/GSTM2/GSTM1/ITGAV/MAP2K4/BMPR1A/GSTO1/MGST3/MAP2K5/RELA/MAPK10/PLAT/ARHGEF2/JUN
hsa05208	Chemical carcinogenesis - reactive oxygen species	PPIF/MGST2/PIK3R3/GSTT2/SLC26A2/MAPK14/GSTM4/SDHC/GSTA1/SOD2/NDUFB3/NDUFA10/GSTM2/GSTM1/NDUFB7/MAP2K4/GSTO1/NRAS/NDUFV2/MGST3/NDUFS6/COX6B1/COX7A2L/RELA/UQCRC2/MAPK10/FOXO3/AKR1A1/CYP1B1/AHR/JUN/CYP1A1
hsa01524	Platinum drug resistance	POLH/APAF1/BCL2/MGST2/PIK3R3/GSTT2/GSTM4/GSTA1/GSTM2/GSTM1/GSTO1/MGST3/BIRC2/SLC31A1/PMAIP1
hsa00480	Glutathione metabolism	RRM2/MGST2/GSTT2/GPX3/GSTM4/GSTA1/GSTM2/GSTM1/GSTO1/MGST3/SRM/GCLC/IDH1
hsa00900	Terpenoid backbone biosynthesis	MVK/HMGCS1/PCYOX1/FDPS/FNTA/PDSS2/GGPS1
hsa04110	Cell cycle	HDAC1/CCND2/CCNE2/TGFB1/CDKN2C/CCND3/TFDP1/YWHAH/CDC7/SKP2/RBL1/CDK2/PCNA/YWHAZ/E2F3/CDC23/ORC4/CCNH/CDC6/GADD45A
hsa05222	Small cell lung cancer	CCNE2/APAF1/BCL2/PIK3R3/SKP2/ITGA6/ITGAV/CDK2/ITGA3/RXRA/E2F3/BIRC2/RELA/DDB2/TRAF1/GADD45A

hsa03040	Spliceosome	HNRNPM/FUS/SRSF3/SF3B3/SRSF1/TRA2A/LSM5/DDX23/ACIN1/TRA2B/DDX46/SNRPF/DHX15/SNRPD3/SNRPD1/PRPF31/SNW1/SRSF4/SYF2/PRPF3/BUD31/SART1

Table 2S

ID	Pathways	Genes
hsa01524	Platinum drug resistance	BCL2/PIK3R3/POLH/GSTT2/APAF1/FADD/GSTM1/GSTA1/PIK3R2/XPA/GSTM2/MGST2/CASP9/MAP3K5/BRCA1/GSTM3/MGST3/GSTO1/SLC31A1/PIK3CA/PMAIP1
hsa04210	Apoptosis	LMNB1/BCL2/CTSO/PIK3R3/TNFSF10/APAF1/FADD/ENDOG/CTSS/PIK3R2/CASP9/CTSC/MAP3K5/LMNA/MAP2K1/MCL1/EIF2S1/PIK3CA/PTPN13/ATF4/PMAIP1/CTSK/JUN/GADD45A
hsa00900	Terpenoid backbone biosynthesis	PMVK/ICMT/FDPS/IDI1/PDSS2/FNTA/HMGCS1/GGPS1
hsa03040	Spliceosome	TRA2A/SRSF5/TRA2B/SRSF1/HNRNPM/SRSF3/LSM5/TXNL4A/NCBP2/LSM4/SNRPF/SNRPC/PRPF31/PRPF38A/HNRNPC/DHX16/BUD31/SF3B1/SART1/SNW1/PRPF3/SYF2/SRSF4/SMNDC1/RBM8A
hsa04068	FoxO signaling pathway	CCND2/MAPK14/PIK3R3/TNFSF10/SGK1/TGFB1/SKP2/PIK3R2/GABARAP/MAP2K1/CREBBP/PIK3CA/SMAD4/EGFR/ATG12/PTEN/S1PR1/CCNG2/BCL6/IRS1/FOXO3/PRMT1/GADD45A
hsa04140	Autophagy - animal	BCL2/PIK3R3/ATG10/ULK2/ATG7/PRKACA/PIK3R2/GABARAP/HMGB1/MAP2K1/ATG14/SMCR8/MTMR3/EIF2S1/PIK3CA/ATG12/PTEN/IGBP1/UVRAG/DDIT4/IRS1/TRAF6/WIPI1/C9orf72
hsa04390	Hippo signaling pathway	FZD1/DLG1/BMP2/CCND2/AJUBA/PPP2R1B/BTRC/YWHAH/PPP1CA/AFP/TGFB1/NF2/TCF7/CCND3/WWTR1/FZD6/YWHAQ/YWHAZ/TP53BP2/DVL1/SMAD4/STK3/DLG4/LATS1/RASSF1
hsa05207	Chemical carcinogenesis - receptor activation	ADCY7/BCL2/PIK3R3/ATF6B/GSTT2/GSTM1/PRKACA/ESR1/CCND3/GSTA1/PIK3R2/GSTM2/MGST2/GNAI2/GSTM3/EPHX1/MGST3/GSTO1/RPS6KA3/MAP2K1/AIP/PIK3CA/EGFR/BCL6/FGF7/ATF4/ATF2/VDR/AHR/JUN/CYP1A1
hsa04110	Cell cycle	ESPL1/CCND2/RBL1/CCNE2/CDKN2C/YWHAH/CDC7/HDAC1/TGFB1/TFDP1/CDC16/ANAPC5/CCND3/SKP2/PCNA/YWHAQ/YWHAZ/CREBBP/SMAD4/CCNH/GADD45A
hsa05225	Hepatocellular carcinoma	FZD1/MET/PIK3R3/GSTT2/SMARCD2/TGFB1/TCF7/GSTM1/GSTA1/PIK3R2/GSTM2/MGST2/FZD6/S

		MARCD1/GSTM3/MGST3/GSTO1/MAP2K1/PIK3CA/TXNRD1/DVL1/SMAD4/EGFR/PTEN/CSNK1A1/GADD45A
hsa05208	Chemical carcinogenesis - reactive oxygen species	SLC26A2/PPIF/MET/MAPK14/PIK3R3/NDUFS2/GSTT2/NDUFB7/GSTM1/SDHC/GSTA1/PIK3R2/GSTM2/MGST2/MAP3K5/PLD1/NDUFV2/GSTM3/EPHX1/NDUFS6/MGST3/GSTO1/MAP2K1/COX7A2L/PIK3CA/PTPN1/EGFR/PTEN/FOXO3/AHR/JUN/CYP1A1
hsa05166	Human T-cell leukemia virus 1 infection	ETS1/DLG1/ESPL1/IL1R1/ADCY7/CCND2/CCNE2/CDKN2C/PIK3R3/ATF6B/TGFB1/CDC16/PRKACA/ANAPC5/CCND3/PIK3R2/IL2RB/FDPS/MAP2K1/CREBBP/PIK3CA/SMAD4/TCF3/PPP3R1/PTEN/RELB/ATF4/ZFP36/ATF2/EGR1/JUN
hsa05418	Fluid shear stress and atherosclerosis	GPC1/IL1R1/MAPK14/BCL2/SDC1/PIK3R3/GSTT2/VCAM1/GSTM1/GSTA1/PIK3R2/GSTM2/MGST2/MAP3K5/GSTM3/MGST3/ITGAV/GSTO1/MAP2K5/PIK3CA/CALML4/JUN
hsa04115	p53 signaling pathway	RRM2/CCND2/BCL2/CCNE2/APAF1/CCND3/CASP9/SIVA1/PTEN/CCNG2/PMAIP1/SIAH1/PERP/GADD45A
hsa04928	Parathyroid hormone synthesis, secretion and action	ADCY7/BCL2/ATF6B/PRKACA/PLD1/GNAI2/MAP2K1/HBEGF/JUND/EGFR/ATF4/SP1/CYP27B1/ATF2/VDR/MEF2D/EGR1/NR4A2
hsa00480	Glutathione metabolism	RRM2/GSTT2/GSTM1/GSTA1/GSTM2/ODC1/MGST2/GSTM3/MGST3/GSTO1/GSR/GCLC
hsa01240	Biosynthesis of cofactors	MAT2A/UROS/GGH/DHFR/BCAT2/GGCX/UMPS/GCH1/CTPS1/MTHFD1L/AK7/NMNAT1/AK1/FECH/NFS1/CPOX/COASY/AK6/HSD17B6/GCLC/COX10/ALAS1/MTHFD2
hsa04114	Oocyte meiosis	ESPL1/ADCY7/MAPK14/CCNE2/PPP2R1B/BTRC/YWHAH/PPP1CA/CDC16/PRKACA/ANAPC5/YWHAQ/PPP2R5C/YWHAZ/RPS6KA3/MAP2K1/PPP2R5B/CALML4/PPP3R1/CPEB3
hsa04310	Wnt signaling pathway	DKK1/FZD1/BAMBI/CCND2/RUVBL1/BTRC/TCF7/PRKACA/SFRP4/CCND3/FZD6/CTNND2/RYK/CACYBP/CREBBP/TBL1X/DVL1/SMAD4/PPP3R1/CSNK2A2/CSNK1A1/SIAH1/GPC4/JUN
hsa05203	Viral carcinogenesis	DLG1/CCND2/RBL1/CCNE2/PIK3R3/YWHAH/ATF6B/HDAC1/TRAF5/PRKACA/CCND3/SKP2/PIK3R2/DDX3X/ATP6V0D2/YWHAQ/YWHAZ/GTF2E2/CREBBP/PIK3CA/DNAJA3/SNW1/SP100/ATF4/UBR4/PMAIP1/ATF2/JUN

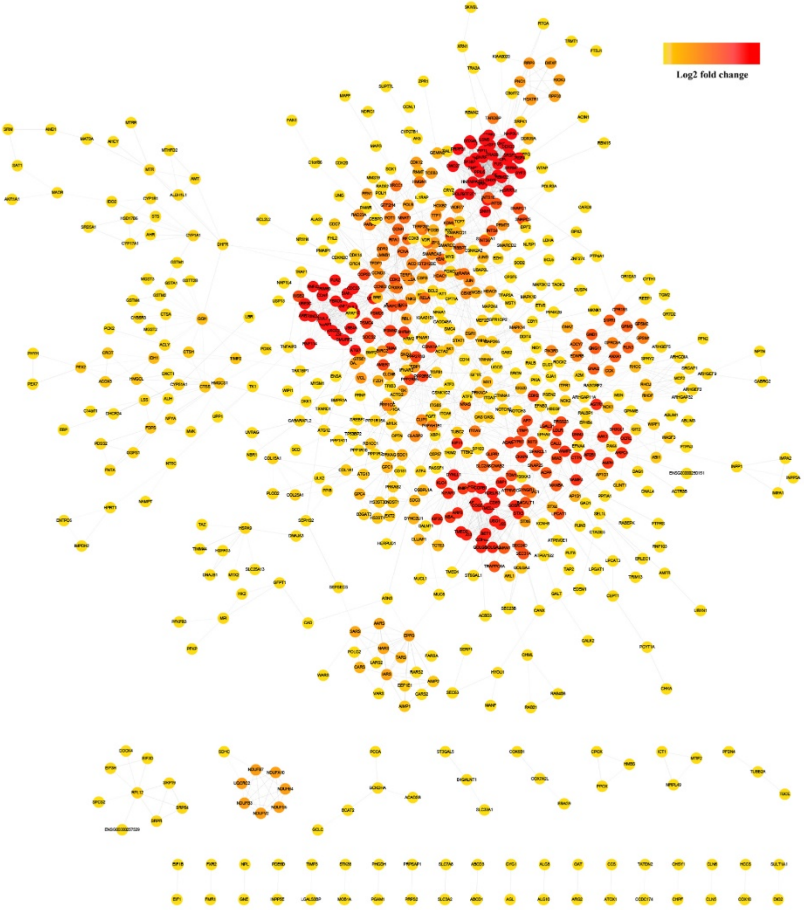

(a)

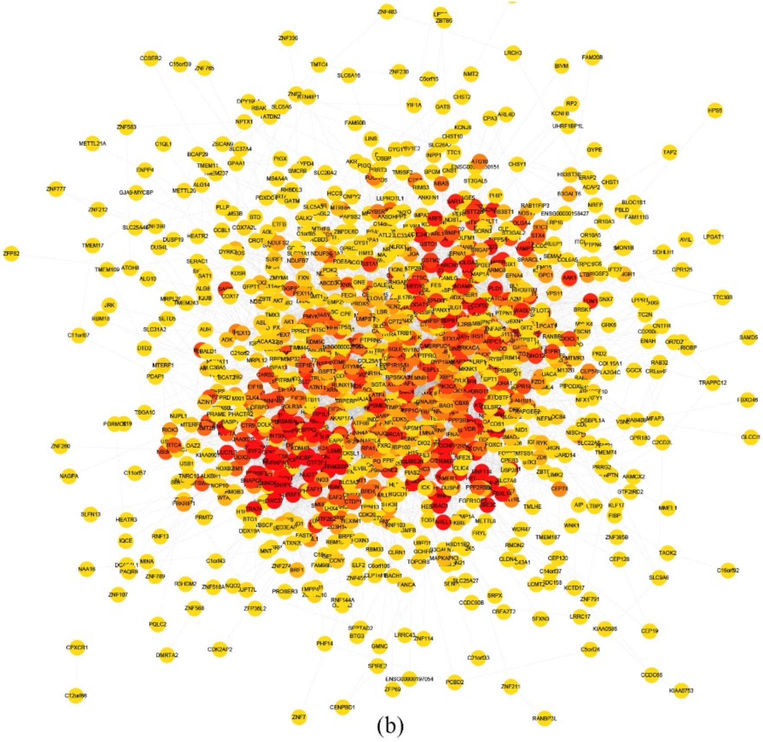

(b)

Figure 2S
